# Bioclimatic transect networks: Powerful observatories of ecological change

**DOI:** 10.1002/ece3.2995

**Published:** 2017-05-19

**Authors:** Stefan Caddy‐Retalic, Alan N. Andersen, Michael J. Aspinwall, Martin F. Breed, Margaret Byrne, Matthew J. Christmas, Ning Dong, Bradley J. Evans, Damien A. Fordham, Greg R. Guerin, Ary A. Hoffmann, Alice C. Hughes, Stephen J. van Leeuwen, Francesca A. McInerney, Suzanne M. Prober, Maurizio Rossetto, Paul D. Rymer, Dorothy A. Steane, Glenda M. Wardle, Andrew J. Lowe

**Affiliations:** ^1^ Australian Transect NetworkTerrestrial Ecosystem Research Network (TERN)AdelaideSAAustralia; ^2^ School of Biological Sciences and Environment InstituteUniversity of AdelaideAdelaideSAAustralia; ^3^ Charles Darwin UniversityDarwinNTAustralia; ^4^ Hawkesbury Institute for the EnvironmentWestern Sydney UniversityParramattaNSWAustralia; ^5^ Science and Conservation DivisionWestern Australian Department of Parks and WildlifeKensingtonWAAustralia; ^6^ Department of Biological SciencesMacquarie UniversityNorth RydeNSWAustralia; ^7^ Ecosystem Modelling and Scaling InfrastructureTerrestrial Ecosystem Research Network (TERN)AdelaideSAAustralia; ^8^ School Life and Environmental SciencesUniversity of SydneySydneyNSWAustralia; ^9^ School of BioSciences, Bio21 InstituteThe University of MelbourneParkvilleVICAustralia; ^10^ Centre for Integrative ConservationXishuangbanna Tropical Botanic GardenChinese Academy of SciencesMenglun, Mengla CountyYunnanChina; ^11^ Sprigg Geobiology Centre and School of Physical SciencesUniversity of AdelaideAdelaideSAAustralia; ^12^ CSIRO Land and WaterWembleyWAAustralia; ^13^ National Herbarium of NSWRoyal Botanic Gardens and Domain TrustSydneyNSWAustralia; ^14^ School of Biological SciencesUniversity of TasmaniaPrivate Bag 55HobartTasmania 7001Australia; ^15^ Faculty of Science, Health, Education and EngineeringUniversity of the Sunshine CoastMaroochydoreQLDAustralia; ^16^ Long Term Ecological Research NetworkTerrestrial Ecosystem Research Network (TERN)AdelaideSAAustralia

**Keywords:** change detection, community turnover, ecological forecasting, environmental gradients, spatial analogues, transect replication

## Abstract

Transects that traverse substantial climate gradients are important tools for climate change research and allow questions on the extent to which phenotypic variation associates with climate, the link between climate and species distributions, and variation in sensitivity to climate change among biomes to be addressed. However, the potential limitations of individual transect studies have recently been highlighted. Here, we argue that replicating and networking transects, along with the introduction of experimental treatments, addresses these concerns. Transect networks provide cost‐effective and robust insights into ecological and evolutionary adaptation and improve forecasting of ecosystem change. We draw on the experience and research facilitated by the Australian Transect Network to demonstrate our case, with examples, to clarify how population‐ and community‐level studies can be integrated with observations from multiple transects, manipulative experiments, genomics, and ecological modeling to gain novel insights into how species and systems respond to climate change. This integration can provide a spatiotemporal understanding of past and future climate‐induced changes, which will inform effective management actions for promoting biodiversity resilience.

## 1 Bioclimatic Transects

Understanding the adaptive potential of species and resilience of communities is vital for effective conservation management in the face of climate change. A particular challenge is scaling up knowledge from detailed local studies to understand ecological dynamics at regional scales. Large‐scale transects that traverse major climate gradients have been recently highlighted as useful platforms for climate change research (de Frenne et al., 2013; Parker, Schile, Vasey, & Callaway, 2011).

**Figure 1:**
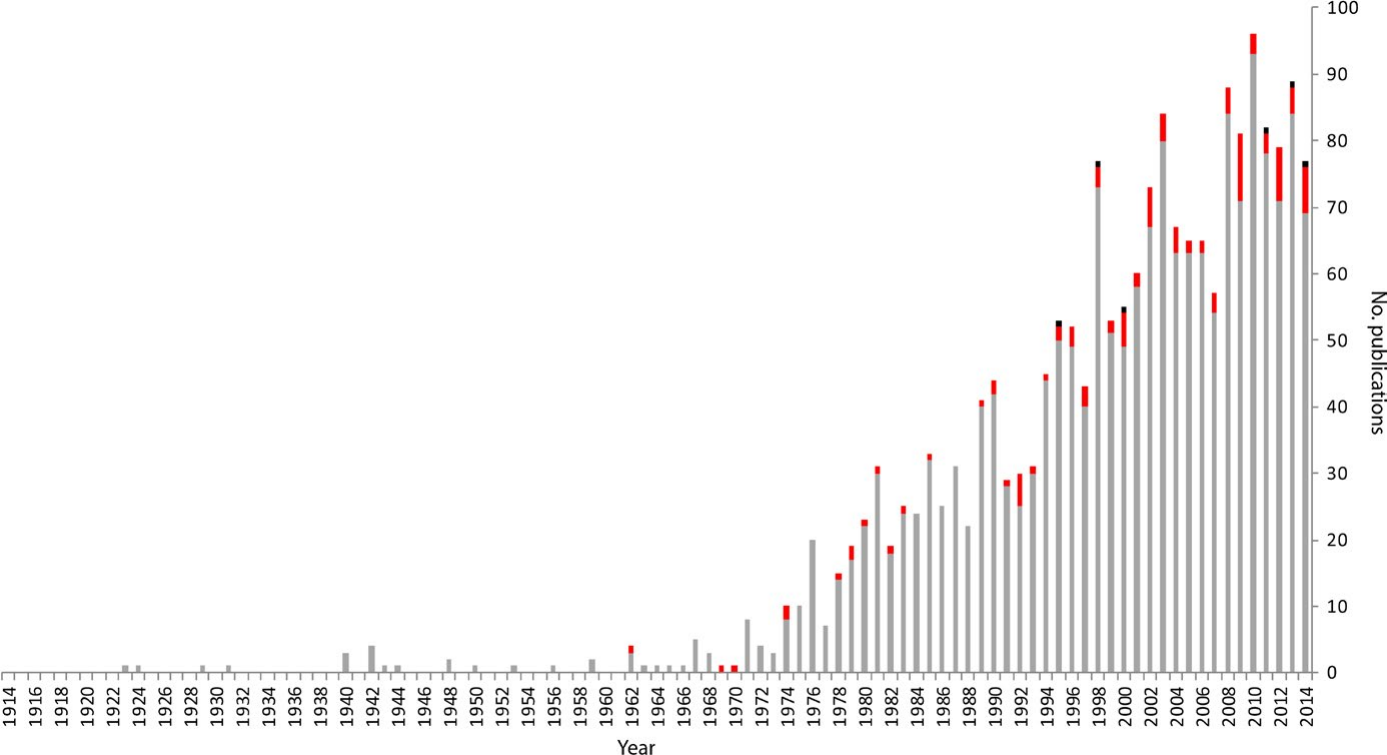
Results from a Web of Science search for peer‐reviewed papers published between 1914 and 2014 containing “transect” in the title in the fields of “environmental science” and “ecology.” Search was undertaken on 12 March 2016. Most studies used single large‐scale transects (e.g., altitudinal or coastal gradients) or several small‐scale transects (e.g., grids for counting birds) (gray bars). A small subset of studies used multiple or replicated transects (e.g., paired altitudinal transects) (red bars). Investigations that included manipulations (e.g., common gardens or translocations) were very rare (black bars)

Bioclimatic transects are a long‐standing method for studying ecological change. By the early 20th century, it was understood that vegetation across Europe and North America responded to a longitudinal rainfall gradient and a latitudinal temperature gradient (Turner, Gardner, & O'Neill, 2001). Whittaker's (1956) classic study of vegetation change in the Smoky Mountains of the United States led to increased interest in environmentally driven biotic change, with a proliferation of large‐scale transect studies since the late 1960s (Figure 1). Two decades later, a global series of subcontinental scale transects was established under the International Geosphere‐Biosphere Program (IGBP) to investigate how climate and land use drive change in ecosystems (Austin & Heyligers, 1991; Koch, Vitousek, Steffen, & Walker, 1995).

Since the IGBP was established, interest in exploring the impacts of climate change on species and ecosystems has resulted in many independent studies using spatial bioclimatic change as a proxy for temporal climate change (Blois, Williams, Fitzpatrick, Jackson, & Ferrier, 2013; Parker et al., 2011). Transects are attractive research platforms because they help stratify environmental variation, reducing the sampling (and therefore resources) required to describe variability (de Frenne et al., 2013), and minimizing confounding factors. They therefore represent a cost‐effective approach for linking biodiversity patterns to environmental drivers in ecosystems (Box 1).

Box 1 **Defining Transects**

1 The term “transect” is used in a broad sense to mean a path (usually linear) through an area along which data are collected. Data collection could include species presence and abundance (e.g., for biodiversity surveys), phenotypic traits, tissue for genetic analysis (e.g., for assessing population genetic structure), and environmental variables. Transects can be used at varying scales: Transects spanning just meters are used as a survey method for measuring vegetation structure within a plot (e.g., White et al., 2012); transects spanning profound environmental change, and potentially along hundreds of kilometers, are more commonly used to assess community composition and adaptive changes along environmental gradients on a large scale (Figure 3, and the focus of this article).

Gillison and Brewer (1985) proposed that positioning a transect to follow a significant environmental gradient was the most efficient method to capture habitat heterogeneity and maximize species detection in biodiversity surveys. This approach differed from traditional survey methods based on random, systematic, or simple stratified sampling (Smartt & Grainger, 1974). Systematic sampling is resource intensive, and Gillison and Brewer criticized randomized sampling as potentially counterproductive, as species' distributions are rarely random. Instead, they proposed that greatest biodiversity would be found in line with the most significant environmental gradient or gradients within a study area, in a nonrandom distribution. They termed these gradient‐orientated transects “gradsects,” which have remained a popular survey methodology (e.g., Austin & Heyligers, 1991; Parker et al., 2011).

Large‐scale (subcontinental) transects follow some gradsect principles. They are placed along a major environmental (often climatic) gradient, site selection is based on logistical considerations (e.g., accessibility), and they follow sound experimental design with opportunities for replication and randomization within a transect. However, where gradsects were designed as a biodiversity survey tool, the goals of bioclimatic gradient studies are typically to assess biotic response to environmental change, and to interpret these results in the context of the gradient.

Transects can be used to examine variation at multiple biological scales, from functional traits and genes within species, to ecosystem turnover, thus providing insights into the relationships between abiotic variables and the adaptive limits of species and communities. Such studies clarify patterns and processes of micro‐ and macro‐evolution, as well as processes that facilitate species persistence and ecosystem resilience, particularly in relation to climate change. Consequently bioclimatic transect research addresses the following fundamental questions:

1. To what extent is phenotypic variation linked to climate, and how much is variation determined by genetics vs. plasticity?
2. What climatic thresholds limit the distribution of species and communities?
3. How do responses to climate change vary among biomes?

Although bioclimatic transects allow for efficient sampling of species and community change across environmental variation, they also have significant limitations (Metz & Tielbörger, 2016; Warren, Cardillo, Rosauer, & Bolnick, 2014). Many environmental variables (e.g., temperature and rainfall) may covary along single transects and so the true driver of biotic change may be difficult to discern (Meirmans, 2015). In addition, species distributions are likely to be strongly influenced by historical factors and not determined solely by contemporary environmental conditions, so current distribution can sometimes be a poor basis for predicting future change (Warren et al., 2014). Results from experiments can be strikingly different from those based on observations over environmental gradients (Metz & Tielbörger, 2016). Thus, caution is required when making predictions based only on contemporary spatial patterning.

**Figure 2:**
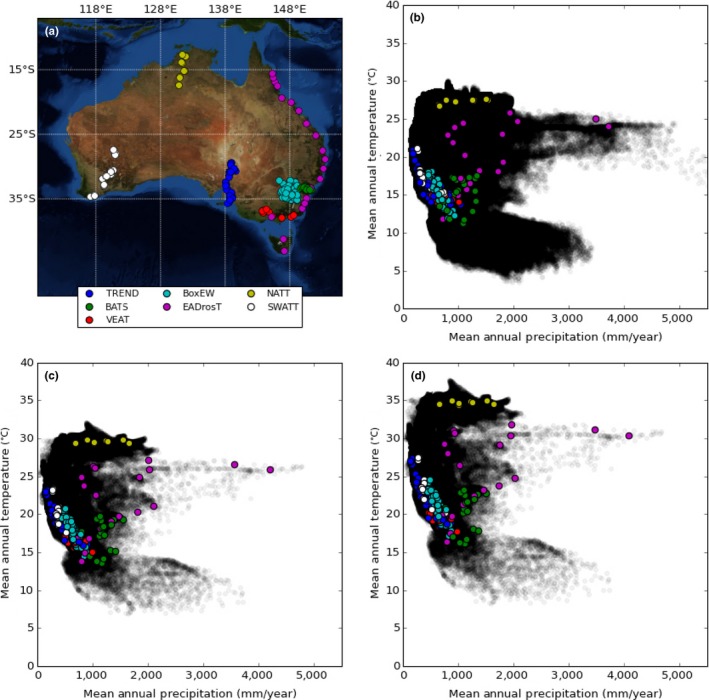
Spatial (a) and bioclimatic (b–d) context of Australian Transect Network sites against recent (1970–2005) and projected (2006–2050) climate space. (b) Recent (1970–2005) ANUClimate v 1.0, 0.01 degree climate data (Hutchinson, Kesteven, & Xu, 2014) mean annual temperature and mean annual precipitation for each site, and all of Australia (gray circles). (c) 2006–2050 ensemble mean of seven global climate models for the RCP4.5 scenario (stabilization of ~650 ppm atmospheric CO_2_ equivalent (Thomson et al., 2011)). (d) 2006–2050 ensemble mean of seven global climate models for the RCP8.5 scenario (comparatively high greenhouse emissions (Riahi et al., 2011)). Models selected to be consistent with current Australian Government climate modeling (CSIRO and Bureau of Meteorology, 2015). Refer to Appendix 1 for details of climate models

Building networks of replicated transects with embedded experiments can address these limitations and help underpin the development of generalized models of how climate affects biodiversity at gene, species, community, and ecosystem levels. In this study, we draw on research facilitated by the Australian Transect Network (ATN; Figure 2; Box 2), a facility of Australia's Terrestrial Ecosystem Research Network, to describe how a network of transect‐based research, augmented by embedded experiments, can overcome the weaknesses of individual transect studies to provide cost‐effective insights into ecological and evolutionary adaptation associated with climate change at the continental scale. Akin to other global networks (e.g., the Pacific‐Asia Biodiversity Transect Network (Mueller‐Dombois & Daehler, 2005)), the ATN has developed a network of bioclimatic transects that cover Australia's major biomes. The ATN straddles most of Australia's climate space and captures the diversity of biomes across the continent. Thus, developing an Australian transect network provides insights that are directly relevant to understanding climate change impacts at multiple scales and provides a framework which could be replicated by other countries wishing to understand the responses of species to changing climates.

Box 2 **Background of the Australian Transect Network (ATN)**

1 The ATN was established as a facility within Australia's Terrestrial Ecosystem Research Network (TERN) with the aim of improving understanding of the climatic drivers and likely future of Australia's biodiversity. The ATN was formed through the development of new and existing transects across several of the major climate transitions in Australia (Figure 2).

**Table:** 

ATN Transects with attributes					
Transect	Gradient	Common metrics			
		Floristics	Focal species	Soil attributes	Indicator species
BATS^a^	170‐km distance 634–1,330 mm MAR^h^ 11.3–17.5°C MAT^i^	Yes	Yes	Yes	Yes
BoxEW^b^	290‐km distance 451–930 mm MAR^h^ 11.8–18.1°C MAT^i^	Yes	Yes	No	No
EADrosT^c^	3,500‐km distance 724–3,719 mm MAR^h^ 11.8–25.8°C MAT^i^	No	Yes	No	Yes
NATT^d^	800‐km distance 640–1,535 mm MAR^h^ 27.0–28.0°C MAT^i^	Yes	No	Yes	Yes
SWATT^e^	900‐km distance 261–746 mm MAR^h^ 15.2–21.2°C MAT^i^	Yes	Yes	Yes	No
TREND^f^	800‐km distance 175–1,049 mm MAR^h^ 13.5–20.9°C MAT^i^	Yes	Yes	Yes	Yes
VEAT^g^	500‐km distance 491–1,018 mm MAR^h^ 13.9–14.9°C MAT^i^	Yes	Yes	Yes	No

It is the vision of the ATN to standardize data collection across all transects to improve multi‐transect analysis. The methodology published by White et al., (2012) is used to ensure data collection and analysis of soils, floristics and indicator species is standardized; with the intention of developing consistent genetic approaches for focal taxa in the future.

**Figure 3:**
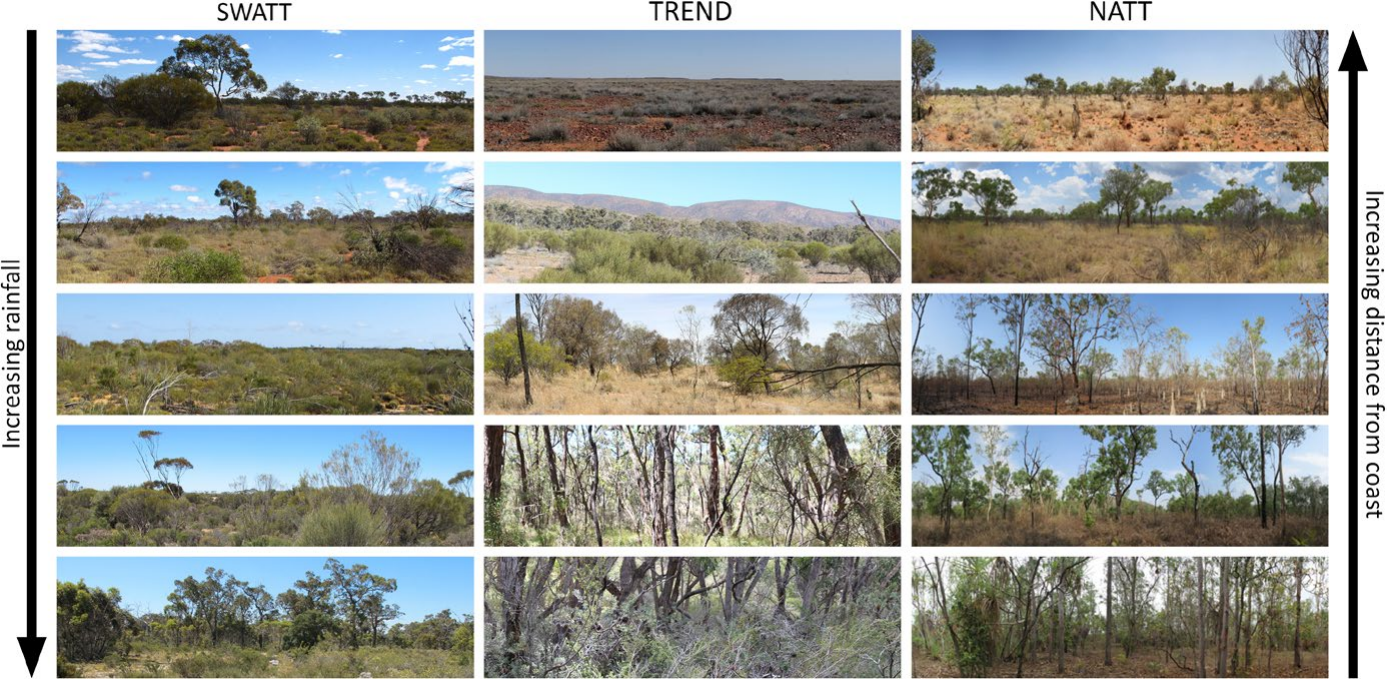
Environmental change across three subcontinental transects

Testing how ecosystems respond to changing conditions is a classic example of transdisciplinary research, which involves researchers and the users of that research collaborating to improve on‐ground conservation outcomes (Campbell et al., 2015). This approach is exemplified by the TREND (Figure 3), which was developed in partnership with the South Australian government's environment agency, and research was tailored to address management driven questions such as “what shifts in distribution, species composition and ecological characteristics can we expect?” (Caddy‐Retalic, Guerin, Sweeney, & Lowe, 2014).

The ATN continues this approach, in part, to provide a platform through which the data and samples collected across several transects can support the ongoing science needs of environmental managers. High‐level questions have been developed to shape the projects supported by the ATN:

1. To what extent can biodiversity be predicted on the basis of environmental variables?
2. Can thresholds be identified where there are abrupt changes in biodiversity?
3. How will ecosystems change in the fact of expected climatic shifts?

Given the dual theoretical and applied interest in answering these questions and potential of transect‐based studies to address them, the development of a continental scale transect network is a powerful approach to understanding and predicting biodiversity change.

Taking globally derived principles, demonstrated using specific case studies from the ATN, we highlight in the study the important insights that can be derived from transect research at both intraspecies (i.e., phenotype and genetic variation) and interspecies (i.e., community) levels, and the importance of combining these two levels. We also summarize key aspects of transect design to mitigate shortcomings of transect methods and highlight the future opportunities provided by such approaches through the application of genomics and modeling approaches. Finally, the continental scope of the ATN provides a model for the establishment of a globally informative network, incorporating variation across the world's major climate zones.

## 2 Insights from Transect Studies

As highlighted above, transect networks provide the opportunity to understand responses to climate variation on multiple scales. Here, we detail how the ATN has provided information at a variety of scales using case studies that illustrate ecological principles and research findings.

### 2.1 Studying Climate Change Within Species

Discounting migration, populations have three main modes of climate change response: (1) plasticity, involving environmental phenotype alteration to increase fitness (Anderson & Gezon, 2015); (2) epigenetics, which improves fitness through the activation and/or deactivation of genes through generations (Heard & Martienssen, 2014); and (3) genetic adaptation, whereby phenotypes adapt over generations through shifts in allele frequencies resulting in improved fitness (Pauls, Nowak, Bálint, & Pfenninger, 2013). Distinguishing between the mechanism(s) underlying apparent responses to climate change (e.g., plastic vs. heritable changes) is often difficult, but is critical for predicting biotic responses to future climate change (Warren et al., 2014).

Identifying causal relationships requires mechanisms to explain relationships between environmental and phenotypic variation (e.g., variation in specific genes, gene expression changes, alteration of chemical pathways, etc.; Savolainen, Lascoux, & Merilä, 2013). Future climatic conditions will probably represent a novel combination of environmental variables; hence, a clear understanding of how changes in climate affect phenotypes is required in order to make predictions of biotic response to future change (Warren et al., 2014).

Phenotypic plasticity and adaptation are often observed as clines in traits that can be related to environmental gradients. For example, potential climatic control over leaf traits has been investigated in the sticky hop bush, *Dodonaea viscosa* (hereafter *Dodonaea*)*. Dodonaea* exhibited clinal variation in leaf area, narrowing with increasing temperature and decreasing rainfall along the along the TRansect for ENvironmental monitoring and Decision making (TREND; Figures 2, 3) in South Australia (Guerin, Wen, & Lowe, 2012). A probable mechanism for this process has been proposed: Leaf narrowing in plants reduces surface area (reducing transpiration and limiting radiation loads), potentially increasing fitness under arid conditions (Guerin et al., 2012). A subsequent analysis of historical herbarium specimens revealed a similar temporal trend: a 40% decrease in leaf width over the last 127 years, with most change occurring since 1950 (Guerin & Lowe, 2013). Whether the phenotypic cline observed in *Dodonaea* is the result of plasticity or genetic adaptation has yet to be determined. However, genomic analysis of this species on the TREND identified 55 genetic variants that strongly associated with temperature and water availability, along with a further 38 genetic variants associated with the elevation of populations (Christmas, Biffin, Breed, & Lowe, 2016a). Many of the variable genes related to environmental stressor responses, such as drought response (Christmas et al., 2016a). These findings suggest that climate is a clear agent of selection pressure on *Dodonaea* populations along TREND and has resulted in local genetic adaptation.

On the same transect, analysis of flowering times of the wallflower orchid, *Diuris orientis*, from herbarium records over the last 100 years identified a shift toward earlier flowering, likely an avoidance response to increasingly arid summers associated with recent climate shifts across its natural range (Macgillivray, Hudson, & Lowe, 2010). A similar phenological change was observed along an altitudinal transect, indicating that ongoing phenological shifts are expected for this species (Macgillivray et al., 2010). These results are consistent with an adaptive response to climate change. The detection of the same trends in both spatial and temporal transects validates the relationship and provides a sound basis on which to seek confirmation through manipulative field and genomic studies.

Rates of adaptation, and thus adaptive potential with climate, are primarily driven be micro‐evolution (i.e., changes in gene frequency) (Visser, 2008). Advances in observing micro‐evolutionary processes of climate adaptation have been made through studying fruit flies (*Drosophila*) along the East Australian Drosophila Transect (EADrosT; Figure 2) (Hoffmann & Weeks, 2007; Rane, Rako, Kapun, Lee, & Hoffmann, 2015). Genetic differentiation among populations has been demonstrated in numerous traits by culturing flies under controlled conditions for multiple generations. Clear differentiation has also been demonstrated in chromosome inversions, specific genes, transposable elements, and maternally inherited bacteria (Hoffmann & Weeks, 2007; Levine, Eckert, & Begun, 2011; Rane et al., 2015). Many of these genetic changes have been shown to be adaptive. For example, cold temperatures led to selection on body size and winter egg retention, and geographic patterns in genetic changes were associated with climate adaptation. Indeed, shifts in gene and inversion clines through time have provided some of the first evidence of adaptive evolution under contemporary climate change (Umina, Weeks, Kearney, McKechnie, & Hoffmann, 2005).

### 2.2 Studying climate responses within ecological communities

**Figure 4:**
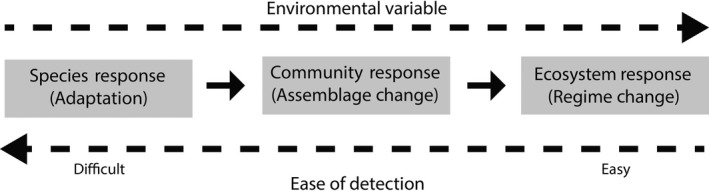
Schematic representation of the hierarchy of ecological change along an environmental gradient. Change progresses from sensitive (but difficult to detect) intraspecific changes in genes or traits (i.e., adaptation), through changes in species assemblage, generally requiring intensive field surveys, to profound (but more readily detectable) biome‐level responses that can be detected using rapid surveys or remote sensing

**Figure 5:**
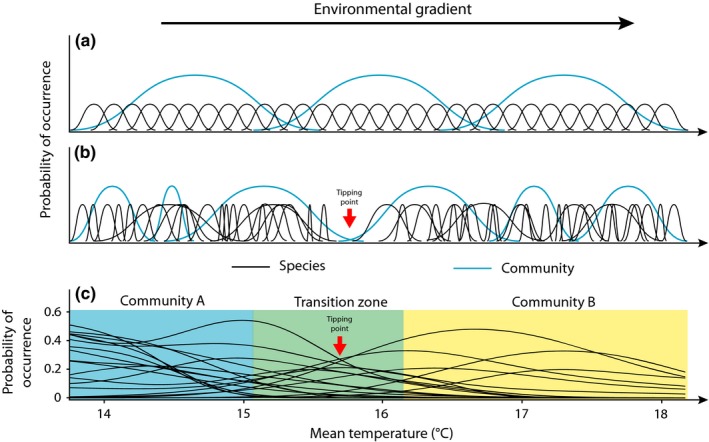
Turnover in species and communities on a hypothetical bioclimatic transect (a, b) and occurrence data from the TRansect for ENvironmental monitoring and Decision making (TREND) in South Australia (c). Regular species turnover would be expected if all species and communities had the same niche width and sensitivity along an even gradient (a). However, landscapes are likely to have a mix of generalist and specialist species with differing tolerances, genetic variation or niche widths, potentially displaying an uneven response between taxonomic and functional groups (b). Red arrows indicate a nonlinear ecological disjunction or “tipping point.” Nonparametric distribution models for 19 common species on the TREND based on surveys of 3,567 field plots by the Biological Survey of South Australia (c). TREND data are provided by the South Australian Department of Environment, Water and Natural Resources, accessed 20 August 2010 (Guerin et al., 2013). Conceptual diagrams after Austin (1985)

When species are pushed beyond their adaptive capacity, some species will be lost and others will shift in space, leading to localized changes in species composition (Figures 4, 5). Measures of species turnover along bioclimatic gradients can provide important insights into how different communities might respond to future climate change. For example, analysis of woody plants along the Northern Australian Tropical Transect (NATT; Figures 2, 3) revealed a systematic decline in species richness with declining rainfall (Bowman, 1996). In contrast, ant species richness was resilient to changes in rainfall, remaining uniformly high across the NATT (Andersen, del Toro, & Parr, 2015). Plant species richness on the South‐West Australian Transitional Transect (SWATT) was positively correlated with rainfall, but beta diversity (spatial turnover) was consistently high at local and regional scales (Gibson, Prober, Meissner, & van Leeuwen, 2017), suggesting species turnover is at least partially driven by neutral processes such as dispersal limitation. Systematic plant community turnover has been observed along the TREND (Figure 5), with families characteristic of mesic ecosystems (e.g., Cyperaceae and Xanthorrhoeaceae) dominating at the temperate end, giving way to a greater prevalence of arid‐adapted families (e.g., Amaranthaceae and Solanaceae) at the drier end (Guerin, Biffin, & Lowe, 2013). Plant community turnover on the SWATT was high and occurred through species replacement (rather than nestedness) across the transect at a local scale, irrespective of environmental factors (Gibson et al., 2017).

Bioclimatic transects are particularly useful for identifying climate‐sensitive zones, where rapid, nonlinear ecological change occurs (Kreyling, Jentsch, & Beier, 2014) (Figure 5). For example, ant species composition along the NATT showed marked discontinuities between the arid and monsoonal zones in the south and between the semi‐arid and mesic zones in the north (Andersen et al., 2015). Along the TREND, particularly rapid species turnover occurred in the range of 15–16**°**C in mean annual temperature and mean annual rainfall of 400–600 mm (Guerin et al., 2013). A similarly abrupt transition from mesic eucalypt woodlands to arid *Acacia* woodlands was detected on the SWATT (Butt, Horwitz, & Mann, 1977). The identification of such climate sensitive zones and biomes is particularly important for conservation planning and prioritization. The transects used in all of these studies has facilitated the stratified collection of biotic and abiotic variables and therefore revealed not only spatiotemporal ecosystem changes, but also the mechanisms responsible for these changes.

Land use (grazing, cropping, reserves, etc.) and intensity can have a major impact on local ecology and can interact with climate to form synergistic effects, particularly if land use changes as a result of climate (Brodie, 2016; Sirami et al., 2016). Transect studies are also useful for investigating community responses to interacting climatic and land use variables. For example, plants at intermittently livestock‐grazed sites across the Box‐gum East‐West Transect (BoxEW; Figure 2) were compositionally more similar to the dry end of the gradient than to ungrazed sites. Characteristic taxa from drier woodlands (e.g., grasses, annual forbs, succulents) become more prominent in grazed mesic woodlands. Conversely, mesic grasses and some perennial forbs that occurred along the whole gradient in ungrazed sites were rare in drier grazed woodlands (Prober & Thiele, 2004). The interaction between community composition and land use history demonstrates the potential for rapid and extensive shifts in plant composition associated with grazing (Prober, Stol, Piper, Gupta, & Cunningham, 2014).

## 3 Strengthening Transect Research

### 3.1 Replication

Deriving causation from analyses of single transects can be problematic. Covariation of many variables (e.g., temperature, rainfall, soil, land use) with geographic distance (Meirmans, 2015) makes it difficult to interpret patterns across single transects, even when manipulative studies are undertaken. Additionally, confounding impacts (such as fire or grazing) occurring on a single transect could be mistaken for a climate signal. Replicating studies along two or more similar gradients helps ameliorate these limitations and filter out confounding factors and enables disassociation of individual drivers, facilitating comparisons of occurrence and variation in genes, and traits between species and communities.

Interpretations of patterns of adaptive change would be strengthened by replicating studies along analogous environmental gradients. Such replicated studies can, for example, help identify whether many genes with small effect, or a few genes with larger effect, provide the basis of adaptive evolution. If the same genes are associated with adaptation across species (and transects), this suggests that there are only a few genetic solutions available to cope with climate change (Bell & Aguirre, 2013; Yeaman et al., 2016). Conversely, if many genes or combinations of genes are adaptive across replicated gradients, there could be substantial flexibility in genetic responses. Studies in three eucalypt species across the SWATT and Victorian *Eucalyptus* Adaptation Transect (VEAT) have demonstrated that adaptation to climate is a genome‐wide phenomenon involving multiple genes and gene pathways in different species (Steane et al., 2017). While there has been extensive discussion around theoretical expectations of the predictability of evolution (e.g., Rockman, 2012), well‐designed transect studies will help resolve this question. Similar investigations of community attributes (such as species diversity) are likely to improve our understanding of community‐level change.

Studies on single transects might identify a correlation between environment and some biotic response, but they are also potentially affected by evolutionary and ecological processes that are disconnected from adaptive processes. For example, habitat fragmentation might limit gene flow and therefore the spread of adaptive genes across a landscape (Breed, Ottewell, Gardner, & Lowe, 2011). Differences between populations might then be interpreted as representing adaptation, whereas they could simply reflect neutral divergence that happens to match an abiotic gradient in a continuous manner (Warren et al., 2014). This problem can be reduced through integrating multiple gradients, such as the elevational and latitudinal sampling approach undertaken on the TREND (Guerin et al., 2012) and EADrosT (Klepsatel, Gáliková, Huber, & Flatt, 2014) (Figure 2). Establishing multiple transects improves characterization of environmental variability, by potentially including multiple gradients running in different directions. In this situation, a single transect would inadequately capture the environmental driver of interest (Travis, Brooker, Clark, & Dytham, 2006). Analyzing data from multiple transects can also disentangle the relative contribution of neutral (e.g., migration—isolation by distance) and adaptive (e.g., selection—isolation by environment) processes to avoid interpreting divergence due to isolation as adaptation (Sexton, Hangartner, & Hoffmann, 2014; Steane et al., 2017).

### 3.2 Embedding experiments

If observations of phenotypic change are repeatedly linked to a climate driver, manipulative experiments (such as reciprocal transplants) and further investigations to identify underlying mechanisms are justified. Transect networks are ideal for such experiments, as independent taxa can be used to determine the generality of biotic responses to climatic drivers. Predictions can then be made as to whether a relationship is likely to persist or change under novel conditions.

Transects provide a robust, cost‐effective platform for investigating phenotypic change through reciprocal transplant experiments, allowing differentiation of plastic and genetic adaptive changes (e.g., Grady et al., 2013; McLean et al., 2014). Indeed, a major focus of many transect research programs is combining growth experiments with genetic data collected along gradients to reveal associations between phenotypic and genetic variation with climate.

This approach has been used to study the red ironbark, *Eucalyptus tricarpa*, and New South Wales waratah, *Telopea speciosissima,* along the VEAT (Figure 2) and Biodiversity and Adaptation Transect Sydney (BATS; Figure 2), respectively (McLean et al., 2014; Rossetto, Thurlby, Offord, Allen, & Weston, 2011; Steane, Potts, McLean, Prober et al., 2014). Local adaptation in functional traits was demonstrated for *E. tricarpa* using common gardens at each end of the VEAT aridity gradient (McLean et al., 2014; Steane, Potts, McLean, Prober et al., 2014). Some traits displayed complex combinations of plasticity and genetic divergence, and several traits showed clinal genetic variation in plasticity itself (McLean et al., 2014).

A combination of genetic adaptation and phenotypic plasticity was also observed in studies of york gum, *Eucalyptus loxophleba*, and gimlet, *Eucalyptus salubris*, on the SWATT (Figures 2, 3) (Prober et al., 2015; Steane, Potts, McLean, Collins 2014). Similarly, studies of *T. speciosissima* along the BATS revealed genetic differentiation of coastal and upland genotypes, with substantial mixing at mid‐elevations (Rossetto et al., 2011). Germination trials showed significant interactions between genotype and germination temperature in growth cabinets and field conditions, where coastal and upland genotypes showed highest germination rates at 30 and 10°C, respectively, suggesting differential selection by optimal germination temperatures in these ecotypes (Rossetto et al., 2011).

### 3.3 Transect network development

The approach of the ATN, IGBP, and other networks in coordinating experiments run by local institutions in ecosystems across continents or globally has become increasingly popular (Fraser et al., 2013). The benefits of coordinated networks are clear: By combining resources and expertise, a consortium can build more than individual researchers, and more reliable results can be obtained by comparing the results from many complementary investigations run simultaneously (Suresh, 2012). Coordinated networks are also able to better target future investment. For example, the ATN is currently focused on ensuring a set of common variables are collected for all transects and embedding experiments on some transects. A major challenge in ensuring the longevity of networks such as the ATN is the availability of centralized funding. If centralized funding is insufficient to support individual researchers and institutions to undertake the work needed to support the network, the function and therefore persistence of the network is quickly jeopardized. Improved long‐term priority setting and funding security for science funding agencies can alleviate this problem.

## 4 New Avenues for Transect Research

Having transect networks available as a research infrastructure resource creates opportunities to apply novel and developing methods to understand species responses to climate change, particularly in the rapidly developing field of genomics and modeling.

### 4.1 Genomics and transectomics

Recent applications of new genomic tools on ATN transects include exploring variation in genome‐wide single nucleotide polymorphisms to understand neutral and adaptive processes in plants (Christmas, Biffin, Breed, & Lowe, 2016b; Steane, Potts, McLean, Prober et al., 2014; Steane et al., 2017) and the nature of genetic changes within chromosomal inversions in *Drosophila* (Rane et al., 2015). Genomic and transcriptomic approaches can test the importance of epigenetics and other modes of gene regulation in natural systems under climate change, which are still not yet well understood (Franks & Hoffmann, 2012), but are likely to be significant (Palumbi, Barshis, Traylor‐Knowles, & Bay, 2014). For example, epigenetic changes have been implicated in drought responses in plants (Rico, Ogaya, Barbeta, & Penuelas, 2014). Transcriptomic studies also indicate that gene regulation is expected to influence phenotypic plasticity and therefore is a likely target of selection (Chen et al., 2012). Experiments to establish causal relationships between molecular changes and trait variation along transects would entail rearing organisms across multiple generations under common conditions to identify epigenetic effects and reciprocal transplants or controlled manipulation experiments to isolate environmental effects. This understanding could facilitate screening for genotypes more resilient to future climates, and assessing benefits of assisted migration for key species (e.g., seed sourcing for restoration programs (Steane, Potts, McLean, Prober et al., 2014; Breed, Stead, Ottewell, Gardner, & Lowe, 2013; Prober et al., 2015)).

### 4.2 Next generation ecological models

Recent advances in forecasting range dynamics and distributions of species have focused on integrating physiological tolerance, adaptive potential, dispersal, metapopulation dynamics, and species interactions (Fordham, Akçakaya, Brook et al., 2013; Fordham, Akçakaya, Araújo et al., 2013; Kearney, Porter, Williams, Ritchie, & Hoffmann, 2009). Transect sampling remains the most efficient way to capture environmentally driven variation across ranges of species and communities (Gillison & Brewer, 1985). Transect networks with wide spatial coverage of bioclimatic space and temporal replication can therefore provide the detailed life‐history data required to parameterize, validate, and refine increasingly realistic ecological models. Physiological and genetic data collected across transect networks can further strengthen model predictions (Fordham, Brook, Moritz, & Nogués‐Bravo, 2014; Wisz et al., 2013; Figure 4). For example, information on physiological adaptation and acclimation to climate variability can be used to modify vital rates in climate‐biodiversity models, improving the reliability of ecological predictions and understanding of eco‐evolutionary dynamics (Thuiller et al., 2013). Resampling transect networks provides opportunities to quantify how species occurrence, abundance and demographic traits vary temporally as well as spatially. Integrating this information into ecological models is important because modeled range dynamics are sensitive to assumptions regarding inter‐annual climate variability (Bateman, Vanderwal, & Johnson, 2012). Building ecological models using transect network data is therefore likely to result in models that more accurately and explicitly reflect species' ecology and responses to changing conditions in both space and time.

## 5 Concluding Remarks

By re‐examining the strengths and limitations of bioclimatic transects for conducting climate change adaptation research, we conclude that a network of bioclimatic transects is a powerful and effective platform to answer the most pressing questions in climate adaptation research. Further understanding of the processes underpinning biotic response to climate change requires manipulative studies that exploit the gradients of change along transects. The case studies illustrate how genetic and phenotypic variation can be linked to improve species distribution models and to forecast changes in biodiversity and ecosystem function. By integrating these approaches into a unified framework, we can improve our understanding of contemporary biodiversity responses to changing climate that will inform effective management actions to promote biodiversity resilience.

## Conflict of Interest

None declared.

## Note

1 Biodiversity and Adaptation Transect Sydney; ^b^Box‐gum transect East–West; ^c^East Australian Drosophila Transect; ^d^Northern Australian Tropical Transect; ^e^Southwest Australian Transitional Transect; ^f^TRansect for ENvironmental monitoring and Decision making; ^g^Victorian *Eucalyptus* Adaptation Transect; ^h^Mean Annual Rainfall (accumulated mm per year); ^i^Mean Annual Temperature (min + max/2).

## Appendix 1 Global Climate Models used in Figure 3

### 1.1

**Table:**

Model	Developer
ACCESS1.0	Bureau of Meteorology, Australia
CESM1‐CAMS	National Center for Atmospheric Research, USA
CNRM‐CM5	Météo‐France & Centre Européen de Recherche et de Formation Avancée en Calcul Scientifique, France
GFDL‐ESM2M	National Oceanic and Atmospheric Administration, USA
HadGEM2‐CC	Met Office, UK
CanESM2	Canadian Centre for Climate Modelling and Analysis, Canada
MIROC5	International Centre for Earth Simulation, Switzerland
NorESM1‐M	Norwegian Climate Centre, Norway

The World Climate Research Programme's Working Group on Coupled Modelling is responsible for the Coupled Model Intercomparison Project, and the climate modeling groups developed the models used in Figure 2. For CMIP the U.S. Department of Energy's Program for Climate Model Diagnosis and Intercomparison provided coordinating support and led development of software infrastructure in partnership with the Global Organization for Earth System Science Portals.

## Appendix 2 Glossary

### 2.1

Adaptation: A heritable change in genotype and/or gene expression in response to environmental change that improves a population's mean fitness.

Adaptive potential: The capacity of a population, species, community, or other biological system to undergo adaptation. Adaptive potential is both facilitated and limited by the levels of standing genetic variation, gene flow, *de novo* mutation, and the inherent plasticity associated with a genotype.

Bioclimatic gradient: A continuous change in one or more climatic variable(s) with associated change in biodiversity. For example: a mesic woodland transitioning to an arid grassland.

Biome: A category of large‐scale ecosystem determined by the structure of the dominant vegetation, such as savanna or tundra. Biomes could comprise a number of constituent ecological communities.

(Ecological) community: An assemblage of organisms that co‐occur and interact in a steady state.

Ecological space: An *n*‐dimensional hypervolume, where *n* represents every variable required for a species' persistence (e.g., sunlight, winter rainfall, food availability).

Epigenetic change: Gene expression moderated by one or more factors external to the gene—such as DNA methylation—that does not alter the gene sequence.

Facilitation: A relationship between two or more organisms conferring an advantage on at least one party. For example, the presence of shading vegetation could create a microhabitat in which smaller plants are able to persist in an otherwise hostile environment.

Functional group: A collection of organisms with shared traits, for example, growth form or climatic requirements.

Functional trait: A trait that is indicative of an organism's interaction with its environment. Functional traits are often governed by balancing fitness trade‐offs in biochemistry and/or physiology. For example, wood‐density is a functional trait of trees that balances growth rate with durability.

Niche: The ecological space in which a species can persist. Generalist species occupy wide niches and are capable of persisting across most (or all) of a climate gradient and might, therefore, display greater adaptive potential. Specialist species occupy narrow niches and could be less likely to persist if environmental conditions change.

Nonlinear change: Change occurring on a gradient associated with one or more tipping points. Nonlinear change could be difficult to model or predict and potentially lead to transformative change within ecosystems.

Phenotypic plasticity: The potential of a genotype to produce variation in phenotype. Variation involves changes in one or more functional trait(s) without changes in gene frequency. Plastic responses can be temporary or permanent for an organism's life. Genotypes vary in their plasticity, and evolution and plastic responses can occur in tandem. Examples include learning or nonheritable changes in gene expression. The mechanisms underlying phenotypic plasticity are not well understood but are likely to involve changes in gene expression in many cases.

Replicated transects: Statistically independent transects traversing similar environmental gradients. Replicated transects can occupy different spatial scales (e.g., a short‐scale altitudinal transect and continental‐scale gradient) but should be otherwise analogous.

Tipping point: The point (in geographic or climate space) at which continuous change in a single environmental factor, or coalescence of multiple factors, reaches a threshold prompting a major ecological disjunction (e.g., a transition from one biome to another).

Tolerance: The ability of an individual, genotype, species, community, or biome to persist in the face of extrinsic change.

Transect network: An arrangement of transects placed across separate environmental gradients on which the same or analogous variables can be measured to develop generalized models of change. Transect networks could include replicated transects as well as transects across different gradients (e.g., aridity, salinity, anthropogenic impact, etc.).
